# It’s Not Irony, it’s Interest Convergence: A CRT Perspective on Racism as Public Health Crisis Statements

**DOI:** 10.1017/jme.2023.10

**Published:** 2022

**Authors:** Tomar Pierson-Brown

**Affiliations:** 1. UNIVERSITY OF PITTSBURGH SCHOOL OF LAW, PITTSBURGH, PA, USA.

**Keywords:** Racism, Public Health, Critical Race Theory, Declarations, Public Policy

## Abstract

Racism as a Public Health Crisis Statements (RPHCs) acknowledge the reality that racism must be eradicated to ensure health justice: a fair and just opportunity for all individuals to be healthy. Scholars of critical race theory (CRT) have expressed doubt when it comes to the capacity of law-related institutions to catalyze or sustain anti-racist efforts. These strains of skepticism underscore the question of whether so many RPHCS were adopted precisely because, in many instances, they were merely symbolic acts. This commentary argues that the trend in adopting RPHCs carries signs of interest convergence, and asserts that the alliance between government and the movement for health justice reflected in this phenomenon falls short of the substantive anti-racist action needed to realize health justice. The spate of RPHC adoption, in lieu of passing anti-racist policy or meaningfully empowering people of color, signifies that the movement for health justice must be strategic in determining whether to leverage, or be wary of, the power dynamics which shape political change. The health justice framework must expand its toolkit to include CRT.

## I. Introduction

Starting in 2014, a range of state and local organizations, from public health associations to County Boards of Commissioners to Governor’s offices, began to issue declarations addressing racism as a public health crisis.^1^ This trend accelerated dramatically during the COVID pandemic, with the adoption of over 200 such statements across the United States, in 2020 alone.^2^ Coinciding with the period of racial reckoning in the U.S. which defined the summer of 2020, the trend of adopting racism as a public health crisis statements (RPHCs) mark an interesting moment in the movement for health justice.

Health Justice is a “jurisprudential and legislative framework” for the eradication of health disparities caused by political subordination.^3^ This framework calls for the courts and other governing bodies to consider and account for the health consequences of their actions.^4^ It also centers engaging and empowering marginalized populations in the development and implementation of health policy.^5^ Advocates may see the trend of RPHCs as a discursive win in the movement for health justice, because naming racism is a crucial first step in dismantling the systems which replicate its harms.^6^ This conversation is happening, in multiple instances, in ways that reflect a sophisticated understanding of what racism is and how its harms translate into health consequences. For example, the RPHC adopted by the Westerville, OH city council defines racism as:

…a social system with multiple dimensions including individual racism that is internalized or interpersonal, covert racism which is subtle and often socially acceptable, overt racism which is blatant and often unrepentant, and systemic racism which is institutional or structural and is a system of structuring opportunity and assigning value based on the social interpretation of value, which unfairly disadvantages specific individuals and communities, while unfairly giving advantages to other individuals and communities and saps the strength of the whole society through the waste of human resources…^7^

This comment argues that the trend in adopting RPHCs carries signs of interest convergence, and asserts that the alliance between government and the movement for health justice reflected in this phenomenon falls short of the substantive anti-racist action needed to realize health justice. Part II provides a deeper definition of interest convergence and identifies the hallmarks of this dynamic. Part III explains that consistent with these characteristics, RPHCs present distinct normative and positive approaches, are attractive for reasons independent of the aims of health justice, and rarely represent the distribution of power. Approaching the trend from this perspective is instructive. Part IV concludes with a discussion of what the movement for health justice can take from the evidence of interest convergence in the adoption of RPHCs. Health justice advocates must utilize CRT and be strategic in determining whether to leverage, or be wary of, the power dynamics which shape political change.

RPHCs are public acknowledgements of a political reality that legal, medical, and public health scholars have been pointing to for over 20 years.^8^ Racism must be targeted as a public health crisis to create a fair and just opportunity for all individuals to be healthy.^9^

While RPHCs correctly name racism as a root cause driving health harms, most have been issued through declarative statements that do not carry any political weight. In response, critics of RPHCs have described them as “menaningless.”^10^ The largely symbolic nature of RPHCs situates the vital discourse on health justice in the gridlock between what it takes to identify a problem and what is required to actually solve it. From this perspective, the trend in RPHC adoption can feel frustratingly ironic. Most RPHCs were released by governmental bodies that possess real political power. These groups could have passed legislation, allocated resources, or imposed consequences in order to disrupt the causal chain between the system of subordination based on the construct of racial identities (racism) and access to the social determinants of health (the resources necessary to realize health justice); but they didn’t. Their decision, to adopt an RPHC rather than public policy that seeks racial equity, suggests that these bodies may have been more interested in appearing aligned with the aims of health justice than in actually realizing it.

When viewed from a critical race theory (CRT) perspective, the irony of only making a symbolic statement that racism is a public health crisis when there was the capacity to take consequential action, is better understood as interest convergence. Interest convergence hypothesizes that anti-subordination efforts will fail unless they are sufficiently aligned with the interests of dominant power. It suggests that the inability of these limited alignments to realize the end-goals of marginalized groups, reflects a passive intent to maintain the status quo.

This comment argues that the trend in adopting RPHCs carries signs of interest convergence, and asserts that the alliance between government and the movement for health justice reflected in this phenomenon falls short of the substantive anti-racist action needed to realize health justice. Part II provides a deeper definition of interest convergence and identifies the hallmarks of this dynamic. Part III explains that consistent with these characteristics, RPHCs present distinct normative and positive approaches, are attractive for reasons independent of the aims of health justice, and rarely represent the distribution of power. Approaching the trend from this perspective is instructive. Part IV concludes with a discussion of what the movement for health justice can take from the evidence of interest convergence in the adoption of RPHCs. Health justice advocates must utilize CRT and be strategic in determining whether to leverage, or be wary of, the power dynamics which shape political change.

## II. Defining Interest Convergence

Under the theory of interest convergence, there will be no racial progress unless the interests of the marginalized align with the interests of those in power. The term “interest convergence” was coined by legal scholar and critical race theorist, Derrick Bell.^11^ Bell’s hypothesis is rooted in the logic that, “Because racism advances the interests of both white elites (materially) and working-class whites (psychically), large segments of society have little incentive to eradicate it.”^12^ Bell is not the only critical race scholar to express doubt when it comes to the capacity of legal structures, as the seat of dominant power, to catalyze or sustain anti-subordination efforts.^13^ In expressing this pessimism, Kenneth Nunn wrote, “African-centered political activity is circumscribed in part because of…law’s limited ability to address issues of concern to African-centered people.”^14^ Peggy Davis described law’s inability to create racial justice because of its inability to hear black voices as part of the collective “we.”^15^

Bell’s premise, that advances in racial justice occur only when white and black interests align, has been criticized as essentialist. Because there is no single set of political goals common to all individuals who identify as either white or black, critics of interest convergence question the theory’s value.^16^ At the same time, however, the logic of interest convergence has been applied to a variety of contexts, including those beyond the black-white racial binary.^17^ This suggests that the provocation has a broad application. Perhaps, this is because the theory has more to say about the nature of power and oppression than it does about a particular (or overgeneralized) racial prerogative.

A further critique of the premise is that the presence of interest convergence is difficult to validate.^18^ In his challenge, Justin Driver argued that the thesis, “…espouses a view of the world that is fundamentally incapable of being falsified by subsequent events.”^19^ Driver has a point: a valid hypothesis should be able to withstand some rigor. Parameters are needed to distinguish instances of self-fulfilling prophesy from those which truly indicate the presence of strange bed-fellows. While it is true that one can never absolutely know what another’s motivations are, evidence of interest convergence can be identified in efforts to address an institutional power imbalance where: (1) there are attractive reasons beyond the aims of justice for addressing the imbalance; (2) the action to address the imbalance presents with distinct normative and positive goals; and (3) the action does not involve the distribution of power to the disenfranchised group.

The first factor is whether there are attractive reasons beyond the aims of justice for addressing the power imbalance at issue. In his essay exploring why the Supreme Court chose to use its decision in *Brown v. Board of Education* to find school segregation unconstitutional, Bell offers two plausible alternative explanations which are distinct from the goals of racial justice. One was to, “… to provide immediate credibility to America’s struggle with communist countries to win the hearts and minds of emerging third world people.”^20^ Likewise, the decision could have been motivated by belief that the southern U.S. would not reach its economic potential unless it was forced to end state-sanctioned segregation.^21^ The real pressures of global politics during the 1950s, and the economy of southern states made these assertions plausible (if unpopular) explanations.^22^ Given that empathy and altruism are motivators for only a few, the presence of incentives beyond the just cause is the first indicator that a stakeholder with power might be taking action based on prevailing interests which converge with those who have less.

The second factor is whether the action taken by the party in power presents distinct positive and normative goals. Normative goals are informed by stories, norms, and narratives. Positive goals are informed by observations. Where the story does not align with actions, there may be evidence of interest convergence. Bell articulates his definition of interest convergence after distinguishing the normative basis for the *Brown* decision (the world ought to reflect racial equality) from the positivist, observable conclusion one might draw about how the world is (“it is clear that racial equality is not deemed legitimate by large segments of the American people…”)^23^ Bell’s observation came from the dissonance between the “hope and promise of *Brown*”, and the development of school desegregation case law in its aftermath.^24^ To be clear, neither normative or positive perspectives are politically neutral. It is the distinction between what an individual or an institution says they are doing and why, and what they are observed to be doing and the consequences, that can be a signifier of the opportunism at the core of interest convergence.

The third factor is whether the action taken in pursuit of justice involves the distribution of power to the subjugated party. The major take-away of Bell’s critique of the significance of *Brown* is that, decades later, racial segregation in schools persists.^25^ In subsequent court cases, Bell points out that the aim of maintaining local control of schools was elevated above the aim of desegregation.^26^ In this way, notwithstanding the *Brown* decision, the power to determine who has access to what sort of public education remained in the hands of local school boards. Moreover, the Courts went on to make proving racial discrimination difficult, and to limit the breadth of relief available in successful claims.^27^ Conservation of power among a privileged party is consistent with the maintenance of the status quo. The failure to follow action with the distribution of power can signal that the impetus for addressing a structural imbalance originated from a goal other than equity.

Taken together, these three factors provide a basis for concluding that an identified outcome has been forged through the power dynamics identified in the interest convergence thesis. The ability to identify the presence of these dynamics is important for health justice advocates. Whether the passage of RPHCs represent a temporary period of overlapping aims, or a catalytic spark which may propel the health justice framework further into the mainstream, can be evaluated in terms of whether or not these actions carry signs of interest convergence.

## III. Evidence of Interest Convergence in the Adoption of RPHCs

Given the power imbalances between those who have the capacity to institute anti-racist policy and those most vulnerable to racism, looking for signs of interest convergence within the adoption of RPHCs can support efforts to make meaning of the trend. Examining both the declarations and the context surrounding them for alternate motivations, differing normative and positive aims, and the distribution of power, reveals evidence of interest convergence, particularly in the adoption of RPHCs by bodies with rule-making authority.

### A. Reasons Beyond the Aims of Health Justice for Addressing Racism as a Public Health Crisis

Beyond the aims of health justice, appeasement and quieting civil unrest are attractive alternative reasons for publicly acknowledging racism as a public health crisis. While some jurisdictions had adopted RPHCs in 2018 and 2019, the number of declarations increased dramatically in 2020 (see Table I).^28^ During this same year, outrage over racism poured into mainstream public opinion in waves. First, came the outcry over the racialization of COVID resulting in attacks on the Asian American community.^29^ This was followed by anger over the disproportionate rates of COVID hospitalizations and deaths in Indigenous, Latin, and African American communities.^30^ Rage escalated following the murders of Ahmaud Arbery, Breonna Taylor, and George Floyd; triggering what has come to be known as the summer of racial reckoning.^31^ The mass pressure to address racial injustice, likely created an incentive for government officials to find low-hanging fruit for quick wins.^32^ Arguably, some of those actions came in the form of adopting RPHCs. The Santa Barbra, CA City Council resolution both declares racism as a public health crisis and condemns police brutality.^33^ Several other declarations name the victims of police brutality in their RPHCs.^34^ A further motivation might come from a kind of peer pressure between groups to adopt their own statements against racism. The RPHCs issued in Pima Co., AZ, and Hennepin Co., MN mention the number of locations across the country that had already adopted statements on racial drivers of health inequities.^35^ Wanting to be on trend with counterparts in other jurisdictions could also account for a decision to adopt an RPHC. These interests provide incentives wholly independent of, yet aligned with, a righteous desire to purse health justice for its own ends. This is the first indicator that interest convergence may have played a role in the adoption of RPHCs.

**Table I:**
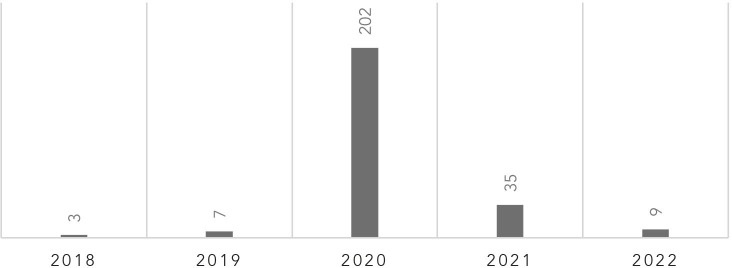
Number of RPHCS Adopted by Year

### B . Distinct Positive and Normative Goals in Addressing Racism as a Public Health Crisis

A subset of the RPHCs passed during or against the backdrop of the U.S.’s most recent racial reckoning, were adopted by governmental bodies with the authority to make rules, allocate resources, and impose consequences for non-compliance. For bodies with this authority, adopting an RPHC instead of passing legislation, revising budgetary schemes, or imposing fines for failure to follow anti-racist policy, presents distinct normative and positive goals. There is a gap between what such groups said they wanted to achieve in their resolutions and what they actually did to bring it about. During its 2021 session, the Oregon Legislature introduced both H.R. 6, a resolution declaring racism to be a public health crisis in the state, and H.B. 2337, a bill that declares racism a public health crisis and requires specific actions meant to reduce racial and ethnic health disparities.^36^ The resolution offers a non-binding statement of the assembly’s intention. The bill provides a mechanism for enforcement and accountability to realize that intention. The resolution (H.R. 6) was adopted, but the bill (H.B. 2337) failed to make it out of committee. There was enough political will to resolve to address racism as a public health crisis, but not enough will to advance the companion legislation. Stating that one’s aim is to reduce health disparities in a manner that carries no capacity to ameliorate the harms, is an action that is inconsistent with stated intention. The second indicator that interest convergence played a role in the adoption of RPHCs can be found where binding action against racism could have been taken, but was not.

A review of 257 RPHCs adopted between 2018 and 2022 indicates that 76% of these statements were passed by a body that has rule-making authority (see Table II).^37^ These bodies include Mayor’s offices, county boards of commissioners, boards of supervisors, state legislatures, and city councils. They have the authority to enact enforceable policy through instruments ranging from executive orders to local ordinances to legislation.^38^ The remainder were passed by bodies that do not have such authority, at least not beyond the confines of their organizations. These groups include school boards, public health associations, boards of health, and non-governmental organizations. For non-rulemaking groups, issuing a public statement may be the fullest expression of their power. If speaking out and raising awareness of the connection between racism and health outcome disparities is the most that an organization can do to move the needle toward change, there is less distinction between the positive observation of speech followed by limited action, and the normative goal of achieving racial justice. The schism between normative and positive aims is greater for those groups which have the authority to enact legislation and enforce orders, but chose only to adopt an RPHC.

**Table 2:**
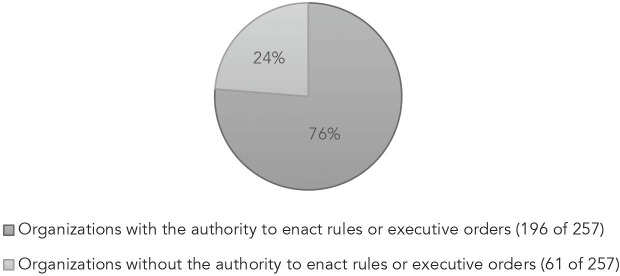
Types of Orgnizations Declaring Racism a Public Health Crisis

Each of the 196 RPHCs that were adopted by a body with rule-making authority could have been introduced as proposed legislation, a new city or county ordinance, or as an executive order. Only nine governing organizations chose to enact enforceable declarations.^39^ The Boston, MA executive order declaring racism a public health crisis, for example, compelled the Mayor’s Office of Health and Human Services to work with the Boston Public Health Commission and other city departments to carry out specific strategies to target health inequities.^40^ Accountability for such RPHCs, comes with the election cycle. A mayor or governor who calls for institutional changes consistent with their order yet fails to deliver, risks the judgment of voters on election day. Enforceable RPHCs demonstrate consistency between normative and positive ends. The work engaged in these jurisdictions illustrates what was possible in addressing the health impact of racism.

In contrast to those RPHCs that align action with stated intention, 95% of the RPHCs adopted by rule making bodies were essentially symbolic, and thus limited in their impact. Ongoing research is needed to determine how many jurisdictions subsequently took steps to implement policy changes consistent with the intentions stated in their declarations. One study, which evaluated select RPHCs according to whether they are actionable, financially responsible to the communities most impacted, and include community participation, among other criteria, provides a model for this kind of research.^41^ While time is one factor that can stand between the articulation of a statement and its realization, the term of office of the individual or body adopting the statement can pose a limitation as well. The RPHC adopted in Colchester, CT in July 2020 by then First Selectman, Mary Bylone, was reversed in November 2021 by the newly elected First Selectman in, “one of the first actions by the town’s new leader on his first day in office.”^42^ The proclamation issued in its place alleged that there are no facts or data to support that racism is a public health crisis affecting the Connecticut town.^43^ As long as there remains divergence between enactment and the action needed to bring conduct in alignment with aspiration, there is an indication that interest convergence was a factor in the adoption of RPHCs.

**Table 3:**
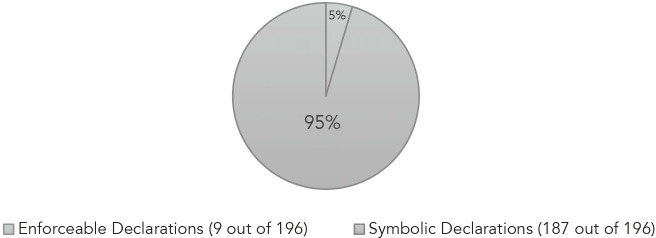
Symbolic Declarations of Racism as a Public Health Crisis

### C . Distribution of Power in Addressing Racism as a Public Health Crisis

Working with the intent to address health inequity, those who adopted RPHCs might have skipped the declaration altogether and simply granted racially marginalized groups the power to lead the development and implementation of anti-racist policy. The RPHC adopted by the Montgomery Co., OH Board of County Commissioners contains a footnote which defines anti-racism as being, “the active process of identifying and eliminating racism by changing systems, organizations, structures, policies and practices and attitudes so that power is redistributed and shared equitably.”^44^ Even as symbolic actions, there was a missed opportunity in the adoption of RPHCs to illustrate and set forth a blueprint for what redistributed and shared power could look like in pursuit of racial and health equity.

There are examples of RPHCs that pledge to share power with communities of color in addressing the health harms caused by racism. The joint statement made by the King Co., WA Executive and the Public Health-Seattle & King County Director, declares their intention to, “share power and resources and work on community-defined problems using community-driven solutions.”^45^ The Washtenaw Co., MI declaration states that its goal of dismantling racist systems includes a commitment to, “sharing power, decision-making and resources with community members who are most impacted by local health disparities…”^46^ Another way that the language of RPHCs might seek to empower racially marginalized groups is by the expressed commitment to centering people of color. The very first action pledged in the RPHC adopted by the city of Fayetteville, AR is to, “center the voices, work, and leadership of the communities most directly affected by said racism.”^47^ Centering efforts demonstrate the primacy of the knowledge and experiences of people of color. It involves shifting the governing viewpoint from, “a majority group’s perspective to that of the marginalized group or groups.”^48^

Empowerment through RPHCs could also occur by directly funding organizations which serve racially marginalized groups. The joint statement issued by Mayor Lightfoot and the Chicago Department of Public Health seeks this means of distributing power, by calling for, “appropriately resourcing groups that are impacted most by racist structures to be key drivers in building and executing solutions.”^49^ Ongoing research is needed to determine how many jurisdictions followed up on their RPHCs through budgetary measures. After adopting its RPHC in 2020, the Lansing City Council then decided to table a proposal from their Mayor to direct money to a new Racial Equity and Anti-Racism Fund.^50^ The proposal would have given $170,000 to local organizations.^51^ In 2021, when the Lansing City Council approved $300,000 dollars to their Mayor’s Racial Justice Plan, none of the funds were identified as going directly to local activists.^52^

Evidence of the missed opportunity to articulate and forge a pathway for the distribution of power directly to racially marginalized groups, whether through access to decision-making, financial resources, or opportunities for consequential participation in problem solving, provides a third basis for concluding that interest convergence played a role in the RPHC trend. Leaving the status quo of authority intact in a document that reads as condemning racism suggests that motivations other than attaining health justice are in play.

Stated commitments to empowering people of color, centering them in the process of addressing racism and health inequity, and affording them access to financial resources, represents the best expressions of power distribution within the language of an RPHC. In contrast, nearly a third of RPHCs (30.35%) used language simply pledging to “engage actively and authentically with communities of color.”^53^ Engagement can be a means of centering. At the same time, given the range of contact that might count as engagement, it could be interpreted so broadly that it minimizes any empowerment that the interaction might convey. While the RPHC adopted in Palm Springs, CA, does declare an intent to expand community power, none of the actions to end racism it commits to (including racial equity training, and the establishment of a Committee for Equity and Social Justice) seeks input from people of color.^54^

Evidence of the missed opportunity to articulate and forge a pathway for the distribution of power directly to racially marginalized groups, whether through access to decision-making, financial resources, or opportunities for consequential participation in problem solving, provides a third basis for concluding that interest convergence played a role in the RPHC trend. Leaving the status quo of authority intact in a document that reads as condemning racism suggests that motivations other than attaining health justice are in play.

## IV. Interest Convergence and the Movement for Health Justice

In many cases, RPHCs present distinct normative and positive approaches, and rarely discuss the need for or the intention to redistribute power. Because there are also attractive reasons for making these statements that differ from the aims of health justice, there is a basis to conclude that interest convergence played a role in this trend. For a window of time, the interests of health justice advocates and certain governmental leaders converged like a Venn diagram; enough overlap for racism to be acknowledged as a public health crisis, but not so much alignment as to institute sustained and substantive anti-racist action.

Finding evidence of interest convergence in the RPHC trend is in no way meant to belittle the efforts of those whose dedicated activism resulted in a local government or public agency’s acknowledgement of the health crisis spawned by racism. It is likely that, in a number of cases, those who adopted RPHCs did so in good faith. They were created by leaders who, on some level, recognized that the health consequences of racism pose a problem and who wanted to do something to address it. The outstanding frustration is that if the desire truly was to bring about a new reality, why — in so many cases — wasn’t the full range of legislative or executive authority applied to effect that outcome? Why didn’t the strategy involve conveying authority or increased resources to people of color? Why not act before the tempest of racial unrest?

Given the evidence that interest convergence has played a role in the trend of adopting RPHCs, there are several lessons that the movement for health justice must take from this moment:

First, interest convergence is a real and consequential political dynamic. Its existence may either be leveraged to advance health justice, or it may be undermining, creating the illusion of anti-racist action while shutting down avenues for sustainable change. The health justice framework must embrace tools that support advocates’ capacity to assess for the presence of interest convergence, in order to determine when to harness this dynamic to reach strategic ends, and when to avoid the diluted messaging that can occur when interests only temporarily align.

Second, the health justice framework must be explicit and intentional about which outcomes will count as wins. As activists, we can get so used to the struggle that any public discussion of health and injustice can feel like progress or forward momentum. This does not serve the movement. Resolutions pass easily because they pacify; they seldom disrupt. There is a risk of regression politics if the passage of resolutions is relied upon to suggest racism has (already) been addressed. The politics of incrementalism too often becomes the politics of concession, especially when it comes to matters of racial inequity. Actions in service to health justice must be systems-informed; incorporating mechanisms for accountability, opportunities for iteration, and centering those most adversely effected by racism.

Finally, CRT must be affirmatively embraced as a tool of the health justice framework.^55^ Just as the application of Bell’s thesis to the passage of RPHCs compels a deeper examination of political incentive and resistance, the tenets of critical race theory offer additional entry points for evaluating and catalyzing progress toward equity. Further scholarship and praxis that draws upon the work of CRT scholars is needed to expand the health justice framework as a call to action; not just for those with legislative and judicial power, but for those seeking to redistribute that power.

The theory of interest convergence lays bare that even passionately sought social justice visions, like those at the heart of the health justice movement, are not universal motivators. Interest convergence may not be the only reason RPHCs were adopted, but as demonstrated in this commentary, it can certainly be counted among the reasons why they were.

## References

[1] According to the American Public Health Association, the first explicit public declaration of racism as a public health crisis was adopted by the Wisconsin Public Health Association (WPHA) on May 22, 2018. See *Racism as a Public Health Crisis: From Declaration to Action*, American Public Health Association, *available at* <https://endingracism.apha.org/#Milwaukee-chapter-1-background> (last visited October 28, 2022). Efforts to address racial equity more broadly date to 2014, when the Dane County, Wisconsin Board of Supervisors introduced and unanimously passed a resolution focused on decreasing racial disparities and increasing equity. See, C. N. Lewis, R. Yearby, C. Gibson, A. R. Jaeger, and M.T. Lawson, *Racism as a Public Health Crisis: How Local Governments are Responding,* The Institute for Healing Justice & Equity (August 2022), *available at* <https://ihje.org/racism-is-a-public-health-crisis-report-2/> (last visited October 28, 2022).

[2] See *Racism is a Public Health Crisis – Map of Declarations*, American Public Health Association, *available at* <https://apha.org/racism-declarations> (last visited October 22, 2022). The data and analysis in this comment is based on the 256 state or local-level resolutions adopted between May 22, 2018 and July 15, 2022, as indicated by the APHA Map of Declarations on July 15, 2022.

[3] E. Benfer , “Health Justice: A Framework (and Call to Action) for the Elimination of Health Inequity and Social Injustice,” American University Law Review 65, no. 2 (2015): 275–351, at 277. 28221739

[4] Benfer, *Id.* at 337.

[5] E. Benfer , S. Mohapatra , L. Wiley , and R. Yearby , “Health Justice Strategies to Combat the Pandemic: Eliminating Discrimination, Poverty, and Health Disparities During and After COVID-19,” Yale Journal of Health Policy, Law, and Ethics 19 (2020): 122–171, 128–9.

[6] See, e.g. *Analysis: Declarations of Racism as a Public Health Crisis*, American Public Health Association, October 2021, *available at* <https://www.apha.org/-/media/Files/PDF/topics/racism/Racism_Declarations_Analysis.ashx> (last visited October 28, 2022); and “The CDC Says Racism Is A Public Health Threat. Here’s What It Means,” National Public Radio, April 9, 2021, *available at* <https://www.npr.org/2021/04/09/985660312/the-cdc-says-racism-is-a-public-health-threat-heres-what-it-means> (last visited October 28, 2022).

[7] City Council, City of Westerville, OH, *Resolution No.* 2020*-10* (June 16, 2020), *available at: <* https://www.morpc.org/wordpress/wp-content/uploads/2020/06/Racism-as-Public-Health-Crisis-Resolution-FINAL.pdf> (last visited October 28, 2022).

[8] See C.P. Jones. “Confronting Institutionalized Racism,” *Phylon* 50, no. ½ (2002): 7–22. Dr. Jones points to 1998 as the year that the public health community first articulated its commitment to eliminating racial health disparities.

[9] P. Braveman, E. Arkin, T. Orleans, D. Proctor, and A. Plough, *What Is Health Equity? And What Difference Does a Definition Make?* Robert Wood Johnson Foundation (May 1, 2017).

[10] See, “Growing List of Cities, Including KCMO, Declare Racism a Public Health Crisis,” *Fox 4 News* (October 5, 2020), *available at* <https://fox4kc.com/news/growing-list-of-cities-including-kcmo-declare-racism-a-public-health-crisis/> (last visited, October 28, 2022) (“Some clergy called the Indianapolis resolution “meaningless.”).

[11] D. Bell , “*Brown v. Board of Education* and the Interest-Convergence Dilemma,” Harvard Law Review 93, no. 3 (1980): 518–533,523.

[12] R. Delgado and J. Stefancic , Critical Race Theory: An Introduction (New York: New York University Press, 2017): at 9.

[13] See, e.g., A.D.P. Cummings , “A Furious Kinship: Critical Race Theory and the Hip Hop Nation,” University of Louisville Law Review 48 (2010): 499–577, 519

[14] K.B. Nunn , “Law as a Eurocentric Enterprise,” Law & Inequality 15, no.2 (1997): 323–371, 365.

[15] P.C. Davis , “Law as Microaggression,” Yale Law Journal 98, no. 8 (1989): 1559–1577, 1573.

[16] See, e.g., J. Driver , “Rethinking the Interest-Convergence Thesis,” Northwestern University Law Review 105 (2011): 149–197, 165.

[17] Driver, Id . at 153–155.

[18] Driver, *Id*. at 181.

[19] *Id*.

[20] See Bell, *supra* note 11, at 524.

[21] *Id.*

[22] Delgado, *supra* note 12, at 23–24.

[23] Bell, *supra* note 11, at 523.

[24] *Id.*

[25] *Id.* at 528. For a more current perspective on the persistence of school segregation, see N. Hannah-Jones, “Choosing a School for My Daughter in a Segregated City,” *New York Times Magazine*, June 9, 2016, *available at* <https://www.nytimes.com/2016/06/12/magazine/choosing-a-school-for-my-daughter-in-a-segregated-city.html> (last visited October 28, 2022).

[26] Bell, *supra* note 11, at 526.

[27] Bell, *supra* note 11, at 527.

[28] Table based on the 256 RPHCs catalogued by the APHA. See, *Racism is a Public Health Crisis – Map of Declarations*, *supra* note 2.

[29] See, Y. Li and H. L. Nicholson Jr. “When “Model Minorities” Become “Yellow Peril”— Othering and the Racialization of Asian Americans in the COVID-19 Pandemic,” Sociology Compass 15, no. 2 (2021):1–13. 10.1111/soc4.12849 PMC7995194 33786062

[30] See, R. Yearby and S. Mohapatra , “Law, Structural Racism, and the COVID-19 Pandemic,” Journal of Law and the Biosciences 7, no. 1 (January-June 2020): 1–20, 2–3. 10.1093/jlb/lsaa036 PMC7313873 32879732

[31] See, A. Chang and R. Martin , *Summer of Racial Reckoning* (August 16, 2020) National Public Radio Website, *available at* <https://www.npr.org/2020/08/16/902179773/summer-of-racial-reckoning-the-match-lit> (last visited October 22, 2022); and N. Chavez, 2020: *The Year America Confronted Racism* (2020), CNN Website, *available at* <https://www.cnn.com/interactive/2020/12/us/america-racism-2020/> (last visited July 28, 2022).

[32] See, K.Y. Taylor, “Did Last Summer’s Black Lives Matter Protests Change Anything?” *New Yorker*, August 6, 2021, *available at* <https://www.newyorker.com/news/our-columnists/did-last-summers-protests-change-anything> (last visited October 28, 2022). The author describes the pressure that the summer of 2020 protests put on then Mayor of Philadelphia to address racism. “True to their sensibilities, elected officials quickly tugged the low-hanging fruit of symbolic transformation.”

[33] See, T. Hayden, “Santa Barbra Declares Racism a Public Health Crisis, Condemns Police Brutality,” *Santa Barbara Independent*, June 24, 2020, *available at* <https://www.independent.com/2020/06/24/santa-barbara-declares-racism-a-public-health-crisis-condemns-police-brutality/> (last visited, October 28, 2022).

[34] See, e.g., *Coachella City Council Resolution No.* 2020*-45* (July 22, 2020), *available at* <https://mccmeetingspublic.blob.core.usgovcloudapi.net/coachelaca-meet-af9017e6c7384516a060b61df95b1661/ITEM-Attachment-001-35290bf599444160b0036120d4912ef2.pdf> (last visited, October 28, 2022); Orange Co., NC Board of Commissioners, *A Resolution Denouncing the Murder of George Floyd and Addressing the Health Director’s Declaration of Structural Racism as a “Public Health Crisis” in Orange County”* (June 2, 2020), *available at* <https://www.orangecountync.gov/DocumentCenter/View/11770/George-Floyd-Resolution> (last visited October 28, 2022).

[35] See, Pima Co., AZ Board of Supervisors, *Resolution Declaring Racial and Ethnic Health Inequities and Income Inequality in Pima County to be a Public Health Crisis*, (December 1, 2020), *available at* <https://webcms.pima.gov/UserFiles/Servers/Server_6/File/Government/Administration/CHHmemosFor%20Web/2020/December/Resolution%20Declaring%20Racial%20and%20Ethnic%20Health%20Inequities%20and%20Income%20Inequality.pdf> (last visited, October 28, 2022); and Hennepin Co., MN Board Action Request No. 20-0242 (June 23, 2020), *available at* <https://hennepin.novusagenda.com/agendapublic/CoverSheet.aspx?ItemID=9340&MeetingID=1024> (last visited October 28, 2022).

[36] *Oregon H.R. 6* (June 30, 2021), *available at* <https://olis.oregonlegislature.gov/liz/2021R1/Measures/Overview/HR0006>; and *Oregon H.B. 2337* (last action June 26, 2021), *available at* <https://olis.oregonlegislature.gov/liz/2021R1/Measures/Overview/HB2337> (last visited October 28, 2022).

[37] As of July 30, 2022, the APHA map of declarations counted 256 RPHCs. It contained a single entry for Appleton, WI. The website does not make clear whether it is referring to the Appleton Health Department or the City of Appleton. To make the distinction between groups that could have acted to target racism through the rule-making process, I am counting the Appleton city’s action separately from that of Appleton Health. This is how I arrived at 257 RPHCs. See, *Racism is a Public Health Crisis – Map of Declarations*, *supra* note 2.

[38] See, M.P. Moore and K.R. Cook , “Executive Order: Strike of a Pen, Law of the Land?” Boston Bar Journal 61, no. 3 (2017), *available at* <https://bostonbarjournal.com/2017/08/09/executive-order-strike-of-a-pen-law-of-the-land/> (last visited October 28, 2022).

[39] See, *Connecticut Substitute Senate Bill No.1* (June 14, 2021), *available at* <https://www.cga.ct.gov/2021/ACT/PA/PDF/2021PA-00035-R00SB-00001-PA.PDF> (last visited October 28, 2022); Office of the Mayor of Louisville, KY, *Executive Order no. 2020-022* (December 1, 2020), *available at* <https://louisvilleky.gov/mayor-greg-fischer/document/declaring-racism-public-health-crisis> (last visited October 28, 2022); Office of the Mayor of Beverly, MA, *An Executive Order Declaring Racism a Public Health Issue in the City of Beverly* (August 24, 2020), *available at* <https://www.beverlyma.gov/DocumentCenter/View/1041/Executive-Order-Declaring-Racism-a-Public-Health-Issue-in-the-City-of-Beverly-PDF> (last visited October 28, 2022); City of Boston, MA, *An Executive Order Declaring Racism an Emergency and Public Health Crisis in the City of Boston* (June 12, 2020), *available at* <https://www.boston.gov/departments/mayors-office/bostons-movement-end-racism> (last visited, April 30, 2022); City of Framingham, MA, *Order No. EO2020-004* (June 16, 2020), *available at* <https://www.framinghamma.gov/DocumentCenter/View/38352/Joint-Order-on-Racism-and-Public-Health> (last visited, October 28, 2022); City of Holyoke, MA, *An Executive Order Declaring Racism and Police Violence as Matters of Public Health Constitute a Public Health Emergency* (June 17, 2020), *available at* <https://web.archive.org/web/20220303021734/https:/storage.googleapis.com/proudcity/holyokema/uploads/2020/06/Executive_Order_6_17.pdf> (last visited October 28, 2022); City of Malden, MA, *Executive Order Declaring Racism a Public Health Crisis* (June 18, 2020), *available at* <https://www.cityofmalden.org/DocumentCenter/View/4067/Local-Executive-Order-Declaring-Racism-a-Public-Health-Crisis---June-2020PDF> (last visited October 28, 2022)); State of Michigan Office of Governor, *Executive Order No. 2020-9* (August 5, 2020), *available at* <https://content.govdelivery.com/attachments/MIEOG/2020/08/05/file_attachments/1511606/ED%202020-9%20Addressing%20Racism%20as%20a%20Public%20Health%20Crisis.pdf> (last visited October 28, 2022); and State of NY, *An Act to Declare Racism a Public Health Crisis and to Establish a Working Group to Promote Racial Equity Throughout the State* (January 26, 2021), *available at* <https://legislation.nysenate.gov/pdf/bills/2021/S2987A> (last visited October 28, 2022).

[40] City of Boston, MA, *supra* note 39.

[41] See, L. Paine , et al., “Declaring Racism a Public Health Crisis in the United States: Cure, Poison, or Both?” Frontiers in Public Health 9 (2021), *available at* <https://www.frontiersin.org/articles/10.3389/fpubh.2021.676784/full> (last visited October 28, 2022) 10.3389/fpubh.2021.676784 PMC8265203 34249843

[42] “Controversy in Colchester: First Selectman Reverses Proclamation Declaring Racism a Public Health Crisis,” NBC News Connecticut (November 17, 2021), *available at* <https://www.nbcconnecticut.com/news/local/controversy-in-colchester-first-selectman-reverses-proclamation-declaring-racism-a-public-health-crisis/2653742/> (last visited October 28, 2022)

[43] *Id*.

[44] See, Montgomery Co., OH Board of County Commissioners, *Resolution No. 20-0759* (June 16, 2020), *available at* <https://www.mcohio.org/government/elected_officials/board_of_county_commissioners/resolutions/resolution_search.cfm> (last visited October 28, 2022).

[45] D. Constantine and P. Hays, “Racism is a Public Health Crisis: The Transformation Starts Here. It Starts With Us,” Public Health Insider (June 11, 2020), *available at* <https://publichealthinsider.com/2020/06/11/racism-is-a-public-health-crisis/> (last visited October 28, 2022).

[46] Washtenaw County Board of Health, *A Resolution Naming Racism as a Public Health Crisis and Confirming Our Collective Commitment to Health Equity in Washtenaw County* (June 30, 2020), *available at* <https://www.washtenaw.org/DocumentCenter/View/17190/Resolution-Racism-is-a-Public-Health-Crisis-June-2020---FINAL> (last visited October 28, 2022).

[47] City of Fayetteville, AR, *A Resolution to Adopt a Resolution Proposed by the Mayor’s African American Advisory Council* (August 19, 2022), *available at* <https://www.nwahomepage.com/wp-content/uploads/sites/90/2020/09/Legislation-Text-3.pdf> (last visited October 28, 2022).

[48] R. R. Hardeman , et al., “Structural Racism and Supporting Black Lives – The Role of Health Professionals,” New England Journal of Medicine 375, no. 22 (December 1, 2016): 2113–2115, 2115, *available at* <https://www.nejm.org/doi/full/10.1056/NEJMp1609535> (last visited October 28, 2022). 27732126 10.1056/NEJMp1609535 PMC5588700

[49] City of Chicago, IL – Mayor’s Press Office, Press Release, *Mayor Lightfoot and Chicago Department of Public Health Jointly Declare Racism a Public Health Crisis in Chicago* (June 17, 2021), *available at* <https://www.chicago.gov/content/dam/city/depts/mayor/Press%20Room/Press%20Releases/2021/June/RacismPublicHealthCrisis.pdf> (last visited October 28, 2022).

[50] S. Lehr, “Lansing City Council Declares Racism a Public Health Crisis, Tables Anti-Racism Fund,” *Lansing State Journal* (June 22, 2020), *available at* <https://www.lansingstatejournal.com/story/news/2020/06/22/lansing-racism-public-health-crisis-anti-racism-fund/3236439001/> (last visited October 28, 2022).

[51] *Id.*

[52] E. Murphy, “Lansing City Council Approves $300,000 for Racial Justice Initiative Among City Workforce,” Fox 47 News (July 30, 2021), *available at* <https://www.fox47news.com/neighborhoods/downtown-old-town-reo-town/lansing-city-council-approves-300-000-for-racial-justice-initiative-among-city-workforce> (last visited October 28, 2022).

[53] I counted 78 of 257 RPHCs which contained this phrase. See, e.g. Virginia House Joint Resolution No. 537 (January 13, 2021), *available at* <https://lis.virginia.gov/cgi-bin/legp604.exe?212+ful+HJ537> (last visited October 28, 2022).

[54] See, The City Council of the City of Palm Springs*, Resolution No. 24792,* (September 17, 2020), *available at* <https://www.palmspringsca.gov/home/showpublisheddocument/76870/637425068118370000> (last visited October 28, 2022).

[55] See, e.g., D. Shek , “Centering Race at the Medical-Legal Partnership in Hawai’i,” University of Miami Race & Social Justice Law Review 10, no. 1 (2019): 109–146, at 116, 125.

